# Remote photoplethysmography for health assessment: a review informed by IntelliProve technology

**DOI:** 10.3389/fdgth.2025.1667423

**Published:** 2026-01-05

**Authors:** Alora Brown, Joeri Tulkens, Maxime Mattelin, Tanguy Sanglet, Brecht Dhuyvetters

**Affiliations:** IntelliProve, Ghent, Belgium

**Keywords:** rPPG, health insights, assessment, physiological biomarkers, IntelliProve, review

## Abstract

**Background:**

Remote photoplethysmography (rPPG) is a non-invasive method that accurately measures clinical biomarkers, including heart rate, respiration rate, heart rate variability, blood pressure and oxygen saturation. The contactless technique relies on standard cameras and ambient light, proving highly accessible and significant for the assessment of general health. Despite its potential, comprehensive research on rPPG applications for health assessment is scarce.

**Objective:**

This review summarizes the current state of knowledge on rPPG health assessments, covering both fundamental physiological monitoring and higher-level health insights. The paper consults the rPPG-based HealthTech company, IntelliProve, as a real-world example to identify relevant outputs that are currently applied in everyday settings.

**Methods:**

A literature review was performed to identify validated physiological biomarkers and emerging health metrics in rPPG research, using Google Scholar, PubMed and Scopus.

**Results:**

The search identified 96 relevant studies, of which 54 directly investigated rPPG-related technologies. The remaining papers provided theoretical context and complementary support relevant to rPPG-based health metrics. Similarly to IntelliProve's approach, several studies combined rPPG with additional inputs to enhance the accuracy of complex health assessments, such as sleep quality evaluation. The review identified well-established health outputs, including heart rate, respiratory rate, heart rate variability, hypertension risk and mental stress detection, as well as exploratory health metrics, including the assessment of mental health risk energy levels, sleep quality and resonant breathing state. To the author's knowledge, existing literature heavily focuses on basic vitals derivation, with limited research into rPPG's broader health applications.

**Conclusions:**

This review synthesizes rPPG-based health applications, demonstrating strong evidence for fundamental physiological monitoring and an increasing interest in higher-level health metrics. Overall, this paper establishes the groundwork for continued research into the growing application of rPPG for health assessments.

## 1 Introduction

### 1.1 Background

Remote photoplethysmography (rPPG) is a revolutionary method for the non-invasive measurement of vital signs (1). It functions through the analysis of recordings, particularly facial videos, providing a low-cost, contactless solution to the collection of physiological parameters. The method relies on the concept that heartbeat pulsations produce fluctuations in blood perfusion in the skin, slightly affecting skin flush. These changes are not visible to the naked eye and require detection by camera-based sensors. Fluctuations in skin color are directly measurable as differences in light absorption changes, occurring as blood within the facial vessels absorb light, and reflect or scatter changes in the surrounding tissues (1, 2). This phenomenon is visible in the red/green/blue (RGB) spectrum, where it is measured and translated into a raw PPG signal, used for the computation of vitals (3). The technique has gained significant traction and attention in recent years due to its potential in accurately assessing vital signs through the use of consumer-grade cameras and without the need for specific expertise and handling (2, 4). It is able to overcome several limitations faced in standard methods, including hygiene-associated drawbacks, as well as prohibitive and restrictive constraints faced by vulnerable populations (1). For example, electrocardiography typically requires a stationary setup, the use of gel patches, chest straps and adherent patches to maintain electrode contact. Not only is this cumbersome to wear for extended periods, but it may also cause skin irritation and allergic reactions. Furthermore, as a non-contact method, rPPG avoids transmission of infection, a valuable feature for risk groups (1, 5). The accuracy of the method has been documented as highly sufficient, with various researchers describing it as comparable to gold-standard practices (2).

As previously discussed in literature, rPPG is widely known to measure the biomarkers heart rate (HR), respiration rate (RR), heart rate variability (HRV), blood pressure (BP) and oxygen saturation (3, 6, 7), proving highly valuable in assessing general health. As opposed to standard methods, rPPG offers a contactless approach, alleviating associated hurdles. The importance of each marker, the limitations associated with its gold standard measurement method, and the respective application of rPPG are discussed below.

#### 1.1.1 Heart rate

HR is the frequency at which the heart beats. It is commonly expressed in beats per minute (bpm). It functions as a key physiological indicator for general well-being, with lower values typically indicating better health (1, 8). HR is traditionally assessed with a PPG sensor, such as a pulse oximeter or an electrocardiogram (ECG). Despite their widespread usage, both systems are limited in that the former is difficult to mount and obstructive to wear for prolonged periods of time, while the latter's requirements to be fixed in place with gel or chest straps can cause irritation and allergy (1). rPPG can measure HR by analyzing the corresponding blood volume signal, and by counting the number of systolic peaks per minute (9).

#### 1.1.2 Heart rate variability

HRV is the variance in time between heart beats. It is a measure of autonomic nervous system (ANS) activity, offering an indirect measure of stress. Stress is associated with increased sympathetic nervous system (SNS) activity, more specifically a withdrawal of parasympathetic influence. The former manifests as an increase in HR, while the latter reduces beat-to-beat variability, thereby decreasing HRV (10, 11). Similarly to HR, HRV is assessed through an ECG which requires long observation periods and is highly sensitive to small inaccuracies. Although ECG is considered a robust technique, it is dependent on the localization of time variations between heart beats, making the aggregation of small inaccuracies prone to distorting results and inducing a measurement bias. Additionally, as previously discussed, the general setup is cumbersome and poorly suited for prolonged usage (3). Although rPPG similarly experiences sensitivity to errors, it provides a fast convenient, contactless alternative to ECG, removing the requirement of electrode placement and time-setup. As in HR, HRV is determined through the pulse signal, by counting the variations in time-intervals between systolic peaks (3).

#### 1.1.3 Respiratory rate

RR is the frequency at which breathing occurs, typically measured in breaths per minute (BPM). It is a vital physiological indicator, giving insight into general health (6). Clinical gold-standard methods of RR measurement include manual counting, telemetry and capnography (12) Each method is limited in that the former is prone to human error (13), and that the latter two are poorly tolerated by patients due to movement-restricting accessories such as electrodes and a nasal cannula, respectively (6, 12). rPPG offers an accurate solution by analyzing the frequency of the derived breathing signals.

#### 1.1.4 Blood pressure

Blood pressure is the force exerted on the walls of the blood vessels by circulating blood, of which high levels can lead to hypertension and chronic inflammation (14). Blood pressure is often measured using a mercury or digital sphygmomanometer, which comprises increasing the relative pressure of an inflatable cuff around patient arms. Although simple, this method may cause discomfort and even pain for some individuals. rPPG can extract a blood volume pressure signal, from which blood pressure can be determined (7).

#### 1.1.5 Oxygen saturation

Oxygen saturation is a measure of the levels of blood oxygenation, assessed by the percentage of haemoglobin bound to oxygen. It is a vital indicator of human health, often acting as a first sign of deterioration. It may be measured by either a pulse oximeter or by more invasive procedures, such as arterial blood gas (1). As discussed, the former method is prone to inconsistency, while the latter requires a draw of blood from the arteries, causing pain and significant risk of infection (15). By analyzing the RGB channel intensities over time, rPPG can determine relative oxygen saturation (1).

### 1.2 Objectives

rPPG shows promise in the field of digital health, however research synthesizing its applications for comprehensive health assessment remains limited. Several studies have validated the application of rPPG for basic physiological monitoring, yet few have systematically reviewed its ability to derive higher-level health metrics, with the brief exception of stress level detection (16) and hypertension prevalence (17).

To address this gap, this paper reviews the current state of knowledge on rPPG-based health assessments, with its scope motivated by the HealthTech company IntelliProve (97). IntelliProve, a rPPG-based health assessment software, was selected considering its breadth of health insights and real-world applications. As a commercially available technology, it outlines the current health applications of rPPG, structuring this review. Accordingly, this review focuses on two categories of health outputs (1) Fundamental biomarkers, namely HR, RR, HRV and a resonant breathing score; and (2) Higher-level health metrics, specifically *hypertension risk, mental stress, mental health risk, energy balance* and *sleep quality*.

To the best of the author's knowledge, this review discusses several exploratory health insights which have yet to be comprehensively discussed in literature. This includes a resonant breathing score, the assessment of sleep quality, the prediction of mental health risk and analysis of a user's energy balance. The review extends the understanding of rPPG applications beyond traditional vital sign monitoring, advancing knowledge on its role in health-assessments.

The remainder of this review is organized as follows. Section 2 outlines the review strategy, inclusion and exclusion criteria, and selection of relevant papers. Section 3 discusses the results, while section 4 contextualizes rPPG by describing its optimal operating principles and potential limitations. It then synthesizes findings from the review, organized according to the health insights implemented by IntelliProve. Each health insight is discussed, highlighting both well-researched, and exploratory metrics. Finally, sections 5 and 6 discuss the review's limitations and conclusions, respectively.

## 2 Methods

### 2.1 Search strategy

This research relied on a literature review to evaluate the current state of knowledge on rPPG and its potential for health assessments. A search was performed in the following databases: Google scholar, PubMed and Scopus. IntelliProve's publicly available documentation was also considered. Additionally, relevant papers were identified by examining the reference lists of the included studies.

### 2.2 Selection criteria

Overall, papers were included if they were written in English, peer reviewed and focused on adult, human studies. Conference papers were also included. A summary of the inclusion and exclusion criteria is listed in Table 1. The search was performed in multiple stages, using a combination of search strings and keywords (Table 2). Multiple targeted searches were performed to address the objectives of this review, each with distinct inclusion criteria. Search one examined the technicalities of rPPG technology (search 1) and its associated limitations. Studies were included if they examined rPPG and limitation-associated topics, such as “distance” and “lighting”. The keywords were selected based on prior research and well-documented drawbacks in the field. Mounting evidence from peer reviewed literature consistently identifies the selected keywords as principal limiting factors in rPPG performance (1, 2, 4). Furthermore, keyword choice was reinforced by the author's firsthand experience and involvement in the field. Search two and three addressed the potential of rPPG in assessing health, including standard vitals (physiological biomarkers) (search 2) and further health insights (search 3). Papers examining both rPPG and PPG were included to expand the search scope, however focus was placed on rPPG. In cases where relevant studies on rPPG were limited, research on PPG was included. For the physiological biomarkers, there had to be an additional focus on one of the following health parameters: heart rate, respiratory rate, heart rate variability and resonant breathing. Concerning further health insights, relevant studies were required to address one of the following: hypertension risk, mental stress, mental health, sleep quality or energy balance. To minimize the exclusion of relevant papers, alternative names for the parameters were included. A final search was employed to address any gaps in the current literature concerning rPPG and health applications. For example, if research was lacking on the applicability of rPPG in evaluating mental stress, papers were selected that analysed the relevance of respiratory rate (a well-documented parameter measurable by rPPG) in assessing stress levels. Inclusion criteria for the final search were less restrictive to allow for a comprehensive assessment. To ensure uniformity in the search, rPPG was defined as a non-contact, remote PPG technique requiring the use of a camera for functionality, while PPG was defined as a contact-based assessment. Additionally, no year-related criteria were applied to ensure comprehensive review.

**Table 1:** A summary of the inclusion and exclusion criteria used to identify eligible studies for this review.

Criterion	Inclusion Criteria	Exclusion Criteria
Language	Written in English	Not written in English
Publication type	Peer-reviewed	Not peer-reviewed (e.g., pre-prints, non-scientific sources)
Study population	Adult human studies	Animal or simulation studies. Studies performed on neonates/minors. Studies in which participants' faces were covered.
Keyword relevance	Containing keywords listed in Table 2, according to the specific search	Did not contain relevant keywords listed in Table 2, according to specific search
Screening process	Passed title, abstract and full-text screening by two reviewers	Excluded at the title/abstract/full text stage for not meeting inclusion criteria

**Table 2:** A list of keywords used in the literature review. Combinations were used to identify relevant studies on rPPG technology, its respective limitations, measurable physiological biomarkers, and further health insights.

Search	Broad Topic	Keywords
1	(Remote) photoplethysmography	(“rPPG” OR “remote photoplethysmography” OR “iPPG” OR “Non contact PPG”) AND (“limitations”) AND (“distance” OR “lighting” OR “short video” OR “ROI” OR “region of interest”)
		(“rPPG” OR “remote photoplethysmography” OR “iPPG” OR “Non contact PPG”) AND (“POS” OR “Plane-Orthogonal-to-Skin”)
2	Physiological biomarkers	(“rPPG” OR “remote photoplethysmography” OR “iPPG” OR “Non contact PPG”) AND (“Physiological parameters” OR “vitals”)
		(“rPPG” OR “remote photoplethysmography” OR “iPPG” OR “Non contact PPG”) AND (“heart rate” OR “pulse rate” OR “respiratory rate” OR “heart rate variability” OR “HRV” OR “resonant breathing” OR “breathing patterns” OR “breathing phase” OR “tidal volume”)
3	Further health insights (Metrics)	(“rPPG” OR “remote photoplethysmography” OR “iPPG” OR “Non contact PPG”) AND (“Hypertension” OR “Hypertension risk” OR “stress” OR “mental stress” OR “mental disorder” OR “mental health” OR “sleep” OR “sleep quality” OR “depression” OR “energy balance” OR “autonomic nervous system” OR “ANS” OR “autonomic regulation” OR “autonomic assessment”)

### 2.3 Screening process

Paper screening was performed in two stages according to the above inclusion criteria. Studies were first screened by title and abstract, followed by an analysis of their entire text. This was to maximize the quality and relevance of the included studies. Quality and relevance in rPPG-related studies were evaluated on methodological rigor and reporting transparency. Studies were required to either be systematic reviews, or original papers. Original papers had to have explicit description of the rPPG setup, including hardware devices and recording conditions. Additionally, the target variable and performance metric had to be transparently defined. Regarding scope, all papers were required to address the terms described in Table 2 and had to meet the criteria outlined in Table 1. Two of the researchers independently screened all papers, with papers included if their eligibility was agreed upon by both reviewers.

## 3 Results

**Figure 1:**
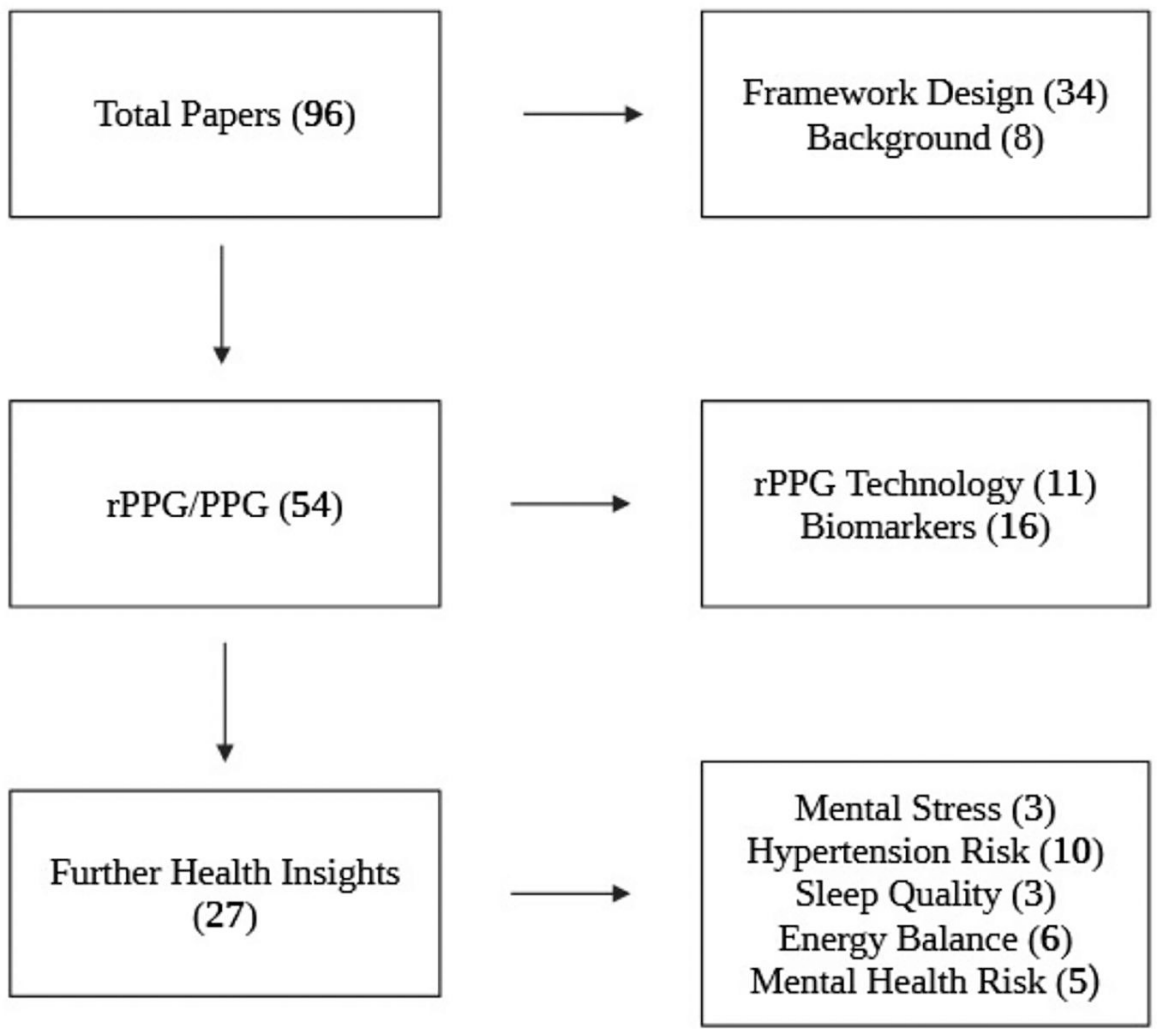
Flow diagram of the included article distribution.

The initial search yielded 16,192 results using “rPPG”, “remote photoplethysmography”, “PPG”, “iPPG” and “Non contact PPG” as foundational search words. Following the inclusion of additional keywords, screening and contingent on the inclusion criteria, 96 papers were included in the final review. Search one yielded 11 results, search two included 16 papers and search three produced 27. A table showing the characteristics of the included studies is found below (Supplementary Table S1) Of the papers, 54 studies directly addressed rPPG and photoplethysmography (PPG), including its technological approach and measurable health insights (Figure 1). The remaining 42 papers were used to provide theoretical context and supporting evidence on the implementation of health metrics relevant to rPPG. The date range is between 1979 and 2025. Interestingly, the studies included in this review are from contemporary research, with 81.4% of the bibliography (*n* = 79) being published between 2015 and 2025. This trend could be explained by a growing interest in applying rPPG for increasingly complex health assessments.

### 3.1 rPPG technology

**Figure 2:**
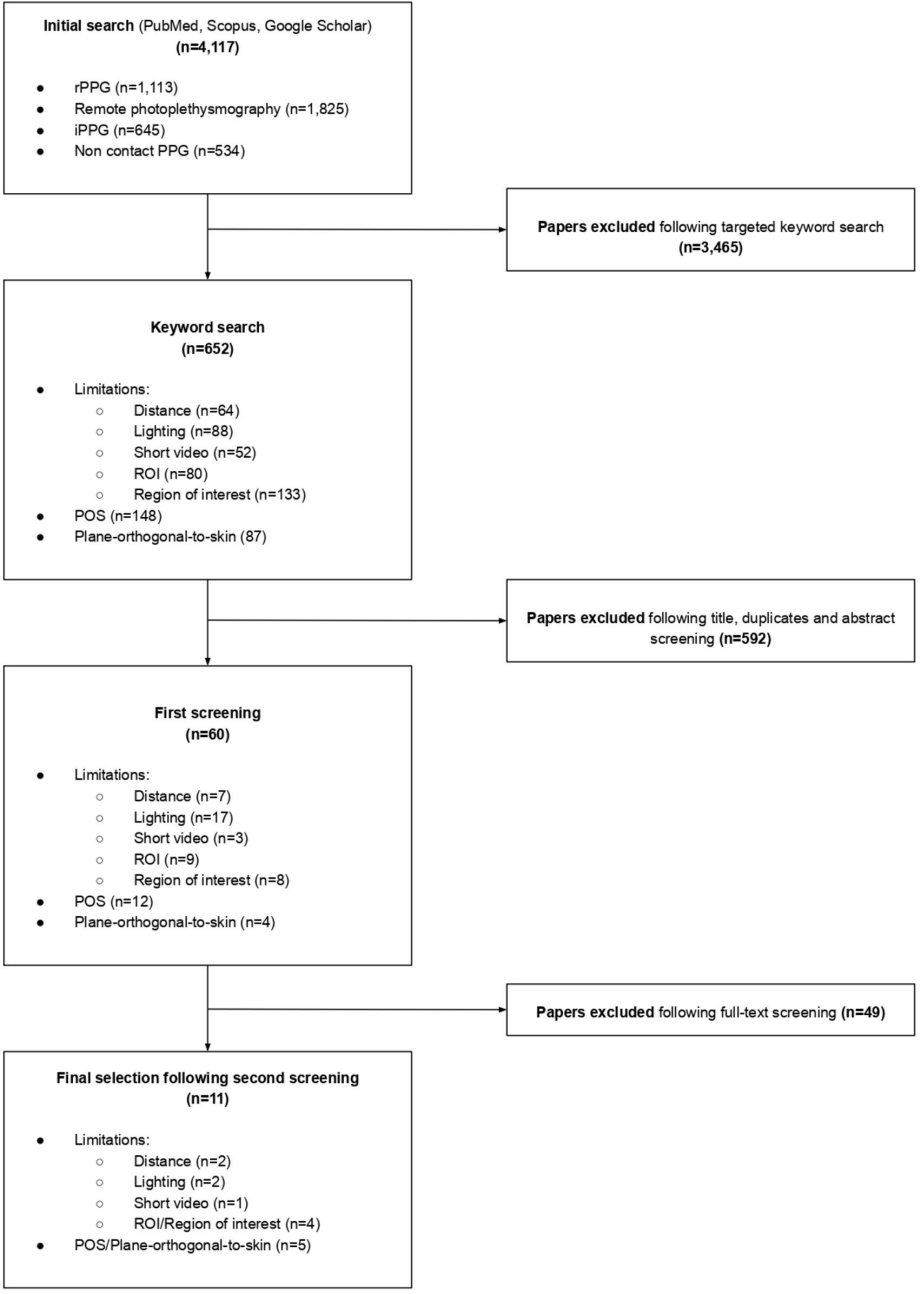
Flowchart depicting the selection process and number of studies included for rPPG technology.

Eleven studies were used to assess the technological methodology of rPPG (Figure 2). There was specific emphasis placed on the POS algorithm. The papers consistently demonstrated POS to be an accurate method for signal processing (20–25). Additionally, several studies examined factors influencing signal quality, finding closer distances to the recording sensor (2, 26), sufficient ambient lighting (2, 21) and shorter video lengths (2) as preferable. The selection of the region of interest (ROI) was also considered by several studies which identified the cheeks, chin and forehead as optimal regions for rPPG measurement (21, 27–29). Literature also identified technological limitations of rPPG, namely variability in skin properties and factors relating to technological set-up (30, 31).

### 3.2 Physiological markers (biomarkers)

**Figure 3:**
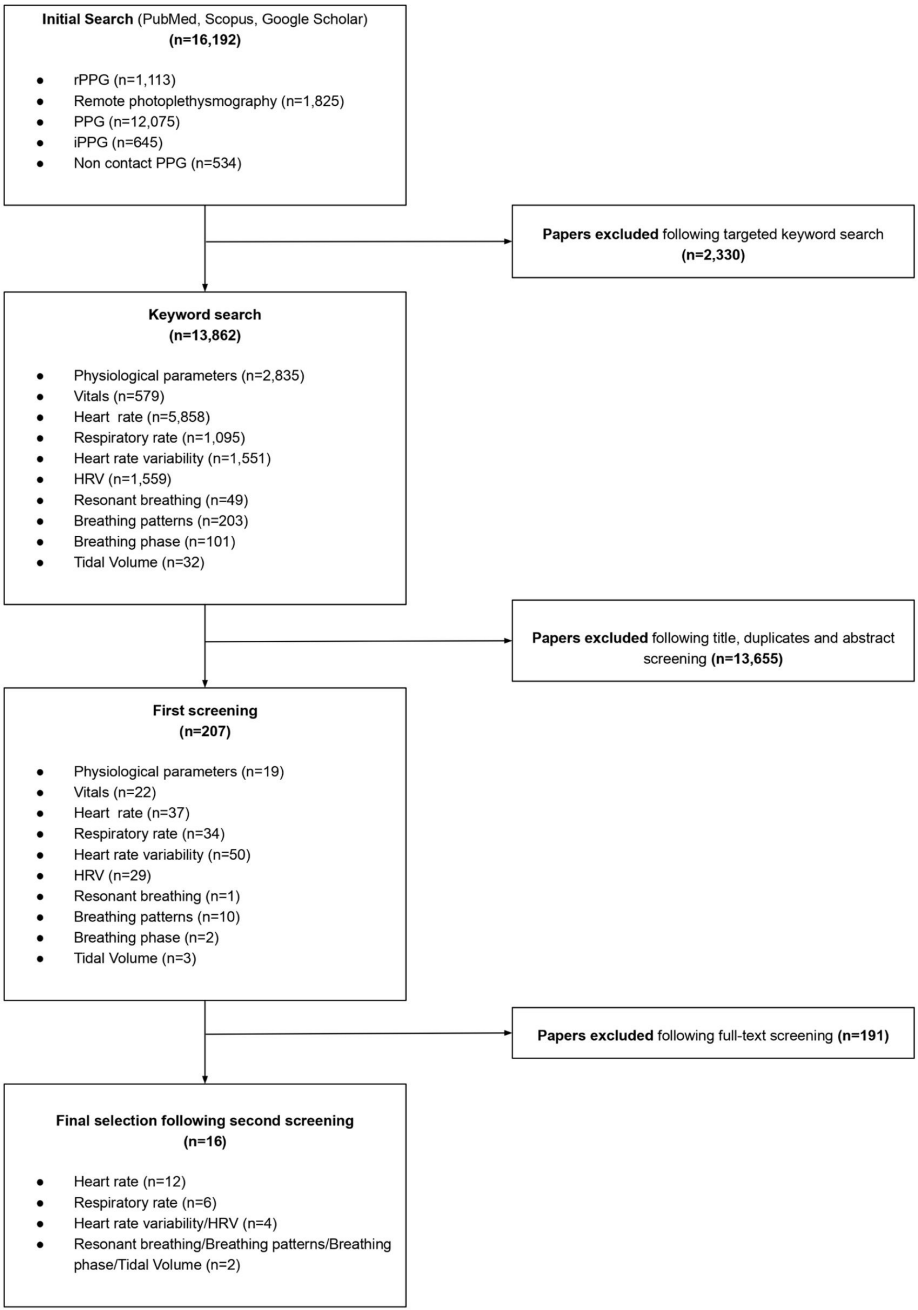
Flowchart depicting the selection process and number of studies included for the physiological markers (biomarkers).

Sixteen studies were selected to evaluate the physiological markers measured by rPPG (Figure 3), of which HR, HRV and RR were identified as physiologically significant and accurately measurable (1–6, 18, 32–37, 91). Following extensive review, two additional studies assessing the applicability of PPG in measuring controlled breathing patterns, including relevant impact on HRV were identified (38, 39). These papers were used in relation to the resonant breathing score.

### 3.3 Further health insights (metrics)

**Figure 4:**
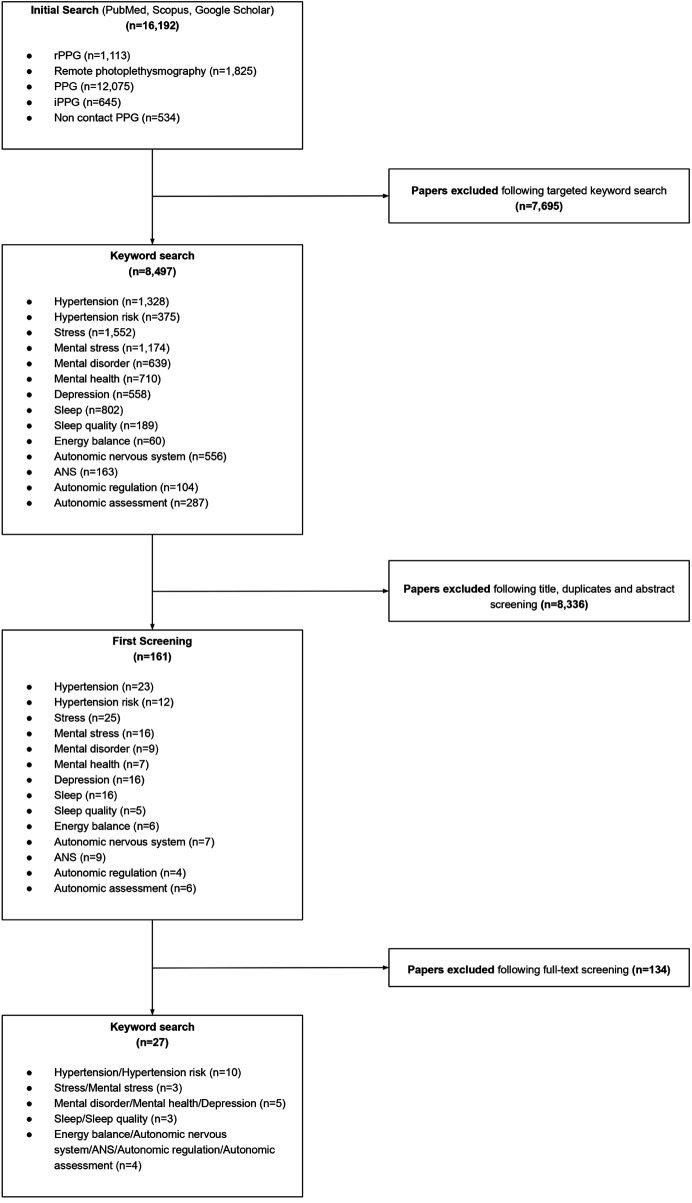
Flowchart depicting the selection process and number of studies included for the further health insights (metrics).

A total of 27 papers were examined and demonstrated rPPG's ability to extract further health insights (Figure 4). Among these, hypertension risk and mental stress were explicitly identified as assessment outcomes. Concerning the remaining metrics, the studies addressed related concepts but did not directly analyze these health insights.

#### 3.3.1 Hypertension risk

Hypertension risk and related concepts were addressed by 10 papers. Of these, eight identified rPPG as a reliable method for blood pressure assessment (17, 32, 40–45), while the remaining two reported PPG's ability to evaluate hypertension risk (46, 47).

#### 3.3.2 Mental stress

Three papers reported the application of rPPG in detecting mental stress. Across these studies, stress was measured through HRV analysis (16, 48, 49).

#### 3.3.3 Mental health risk

Following extensive literature review, no studies assessing mental health risk using rPPG were identified. However, five papers utilizing rPPG (50, 51) and PPG as a method for depression detection were found (52–54).

#### 3.3.4 Energy balance

The literature review was unable to identify studies which explicitly assess energy balance with rPPG. Instead, it focused on the assessment of the autonomic nervous system (ANS), and its respective balance. Accordingly, four studies were identified which utilized rPPG (24, 55) and PPG (56, 57) for ANS evaluation. Throughout these studies and additional relevant research, HR and HRV were reported as accurate methods of measurement (91, 92).

#### 3.3.5 Sleep quality

No studies identifying sleep quality as an rPPG health assessment were identified. However, three papers analyzing rPPG and PPG for sleep related measurements were evaluated. Two studies applied rPPG (58) and PPG (59) for sleep stage evaluation, while one study detected driver fatigue (60).

## 4 Discussion

This section discusses the findings of the review, identifying and analysing the proposed health insights. It also contextualises rPPG technology, outlining its optimal methodology. Combined, these findings provide a foundation for future research into rPPG-based health assessments, by defining a methodological foundation and applications.

### 4.1 Principal findings

In accordance with previous literature, the physiological markers HR, RR, blood pressure and HRV are readily measurable by rPPG, whereas research on further health applications is emerging, but scarce. Previous research has explored the application of rPPG for hypertension risk and stress level detection, however comprehensive insights into mental health risk, energy balance and sleep quality were yet to be systematically discussed. The review revealed an underexplored physiological biomarker, namely the resonant breathing score to assess relaxation state. Furthermore, the combination of rPPG measurements, with additional inputs could enhance the accuracy and reliability of further health insights. In this review, accuracy refers to the proportion of cases in which rPPG-derived measurements are correctly classified relative to the reference device. The definition of a correct classification varies across the included studies, either with the measurement falling within a clinically defined threshold, or reflecting the agreement with a ground-truth label. This review identified a gap in rPPG literature, namely a lack of previous studies synthesising the technology's potential in performing health assessments. As such, the reported accuracy for each physiological measurement and health insight is uneven, as reported in Table 3. This inconsistency highlights a major finding of the paper, namely that research on valuable rPPG metrics is limited. Specifically, to the author's knowledge, the literature review revealed no previous studies that specifically addressed the application of rPPG for the resonant breathing score, mental health risk, energy balance and sleep quality. However, several studies did demonstrate the promising potential of rPPG and explored related aspects of these health metrics. rPPG shows revolutionary potential in the digital health field, with an ability to assess health outcomes remotely and accurately. Not only would this improve general accessibility to well-being evaluation, but it may empower individuals to proactively take charge of their health. Evidently, rPPG presents advantages to the general population, however extended research on its applications remains limited. This review seeks to advance the understanding of its potential and applicability, highlighting the need for continued research in this emerging field.

**Table 3:** Overview of the reported (r)PPG accuracy for each biomarker and metric.

Parameter	Health Parameter	Accuracy
Biomarkers	Heart Rate	99.1% for values ≤ 101 bpm compared to a pulse oximeter (32)
	Respiratory Rate	96% (±6.9 BPM) relative to a chest belt sensor (6)
	Heart Rate Variability	0.9 (Pearson Correlation) (91)
	Resonant Breathing Score	N/A
Metrics	Hypertension Risk	92.31% (F1 score) (46)
	Mental Stress	95.83% compared to estimation from a PPG device (16)
	Mental Health Risk	N/A
	Energy Balance	N/A
	Sleep Quality	N/A

### 4.2 Comparison with prior work

#### 4.2.1 rPPG methodological principles

The methodological principles underlying rPPG are discussed below, with an emphasis on the optimal techniques for signal acquisition and processing. Analysing these techniques is essential for achieving robust health assessments.

The optimal methods of rPPG are briefly described below. First, the processing engine is exposed to a dataset. Typically, participants are instructed to sit within a specified distance of the rPPG-based device, in a well-lit environment. rPPG is deemed a highly accurate method for physiological measurement, considering certain criteria are met, including close distance to the respective sensor and sufficient video length. Rao et al. (26) recommends a distance of less than one meter, while Di Lernia et al. (2) proposes video lengths of 25 s, at sufficiently close distances. Natural, sufficient lighting conditions are also preferred to maintain accuracy in measurements (21).

Following the set-up, facial extraction takes place, corresponding to the identification of the image relating to the face. This process is repeated per frame. Next, the ROI is processed, which based on literature, is optimal in the cheeks, chin, and forehead. These regions are preferential based on their ability to better influence the reflected light due to blood volume changes (21, 29). Notably Bondarenko et al. (29) conducted an analysis on 70 studies, concluding the superiority of multiple, well-distributed ROIs, specifically the cheeks and forehead, to improve rPPG measurement accuracy and robustness. Additionally, research has shown these areas to be the most promising for rPPG function (27–29). Huagg et al. (21) further consolidates the usage of multiple, larger ROIs to increase the overall surface area, further enhancing results. ROI processing includes the collection of pixels per colour channel, which are averaged to produce a pair of RGB signals for each ROI. These are averaged to obtain a single pair of “face RGB signals”.

Before the final rPPG signal is obtained, it typically undergoes preprocessing to ensure that the signal remains clean and relevant (22). Lastly, a rPPG method is applied, with literature reporting plane orthogonal-to-skin (POS) to be the superior technique (23, 24). This method uses a skin-tone perpendicular plane in the RGB signal, and has been reported as highly beneficial for filtering out noise caused by light reflection (20–25). Notably, Momeni et al. (25) applied the POS rPPG approach to measure heart rate in critical care and emergency patients, achieving a mean error fewer than 2 BPM, highlighting the technique's advantage in yielding a low error rate. Additionally, literature has discussed this method as highly reliable when compared to alternative rPPG techniques, with an ability to cope with difficult datasets (21).

#### 4.2.2 Physiological markers (biomarkers)

As discussed, heart rate, heart rate variability, blood pressure, and respiration rate are key physiological biomarkers, successfully measured with rPPG. As seen in previous studies, the three former biomarkers are computed by analysing a blood volume pulse signal, while the latter by a respiration signal. The accuracy of these measurements has been widely validated. Notably, Zuccotti et al. (32) demonstrated high reliability for heart rate, using their non-contact mobile app, achieving a MAE as low as 2.96 BPM and a classification accuracy of 99.1%, suggesting that in 99.1% of cases the app correctly classified HR as ≤101 bpm or >101 bpm compared to a pulse oximeter. The study was conducted on 562 participants, highlighting the robustness of the above results. Additionally, the app demonstrated similar accuracy for its SPO2 measurement, reinforcing the applicability of rPPG for health assessments. Similarly, Shoushan et al. (33) was able to derive rPPG based HR measurements from smartphone and laptop cameras with 99.4% classification accuracy relative to ECG-based ground truth values. rPPG measured respiratory rate has shown similar success, with studies reaching absolute mean errors as low as 0.40 ± 0.11 BPM (34), and accuracy levels as high as 96% (±6.9 BPM) relative to a chest belt sensor (6). Furthermore, several researchers have consolidated the credibility of the measured biomarkers (1–6, 18, 32–38), emphasising their relevance and applicability. HRV has also demonstrated high accuracy in several studies (3, 18, 36, 92), with researchers reporting MAE values as low as 10.5 ms and 6.15 ms (92). Notably, Odinaev et al. (91) found high correlation between finger pulse oximetry and rPPG-extracted HRV at 0.9, demonstrating strong agreement and reliability. Furthermore, HR and RR can be applied to derive averages of the resting heart rate and resting respiratory rate. In both cases, the advanced metrics require the mean of a minimum of four measurements, providing an average reference to HR. Literature describes a minimum of four values to ensure that the output is reliable and significant, compared to that of a single measurement (61). Generally, a lower resting heart rate and resting respiratory rate are indicators of good general health (6, 62).

The resonant breathing score is a measure of how relaxed an individual is while performing a breathing exercise, characterised by the synchronisation of the cardiovascular and respiratory systems (63). The literature review revealed a lack of studies concerning the applicability of rPPG in measuring resonant breathing during relevant exercises. To address this gap, the search scope was expanded to include the ability of PPG in measuring deep breathing patterns (38, 39). Romero et al. (38) successfully measured breathing phases and tidal volume at a breathing rate of 8 BPM, with a mean average error of 0.48 s during inhalation and 0.14 s during exhalation. Additionally, Jan et al. (39) found that PPG was able to track and identify the impact of slow, deep breathing at 5 BPM on HRV. Although these studies do not specifically address rPPG in the context of resonant breathing, they establish precedent in its applicability. Furthermore, considering the close relationship between resonant breathing, slow and deep breathing, and its impact on the cardiovascular system, these findings show relevance and support the application of rPPG-derived resonant breathing scores.

Resonant breathing score can be obtained by instructing participants to slow their breathing pace down to the established resonant pace of six breaths per minute (63), achieved by breathing in and out at 5 s intervals. Following instruction, the rPPG algorithm detects patterns by visualisation from the extracted breathing signals. Additionally, synchronisation to the cardiovascular system causes the pulse signal waveform to adopt a similar morphology. Both waves express a frequency of 0.1 Hz, a phenomenon previously described in literature (63, 64). Resonant breathing has been shown to increase HRV, thereby improving an individual's mental well-being, resilience and adaptability to stress. Research has also documented the practice of slow breathing rate as an effective treatment for stress related disease and ANS dysfunction, emphasising the clinical relevance and importance of this biomarker (63, 64).

#### 4.2.3 Further health insights (metrics)

The discussed physiological parameters form a strong foundation for the screening of general health, and are often leveraged to extract deeper health analytics. Additionally, several studies combine rPPG with additional inputs (e.g.,: facial key-points) to deliver a holistic and accurate measure of health.

##### 4.2.3.1 Hypertension risk

The hypertension risk refers to the probability that an individual will develop consistently elevated blood pressure within the next year. Hypertension refers to increased systolic and diastolic blood pressure readings, of ≥140 mmHg and ≥90 mmHg, respectively. Risk prediction estimates the likelihood that a person currently experiencing normotension (systolic readings < 120 mmHg, diastolic readings<80 mmHg) bypasses these thresholds (65). This metric primarily serves as a probabilistic estimate of health, rather than a diagnosis. Considering blood pressure is defined as the amount of force exerted on the blood vessels by the circulatory system, it is a reflection on the workings of the cardiovascular system, including the force required for function, serving as an indicator of well-being. The application of rPPG for blood pressure estimation and prediction has been explored by several researchers (32, 40–45). Notably, Cheng et al. (40) was able to predict systolic and diastolic blood pressure with a mean average error of 8.721 and 8.653, respectively. Furthermore, Park et al. (45) was able to robustly measure both blood pressure parameters in diverse environments, including outdoors, in moving cars and in flying drones, demonstrating the technology's ability in suiting everyday environments. In regard to hypertension risk, Curran et al. (17) further confirms rPPG as a possible technique to estimate blood pressure, measuring similar values to standard, finger-based PPG, however the study also concludes the possibility of a risk prediction model by comparing blood pressure values to baseline values. This would suggest the measurement of hypertension risk as a credible and valuable insight to derive from rPPG technology. Additionally, the highly comparable method of PPG has demonstrated high accuracy in assessing hypertension risk (46, 47). Liang et al. (46) was able to achieve an F1 score of 92.31% and a specificity of 88.57%, highlighting the health insight's potential.

In addition to rPPG measured blood pressure (systolic and diastolic), the variables age, sex, BMI, smoking status and parental hypertension prevalence are often used to compute hypertension risk. Research has shown the selected inputs to be highly significant, with age naturally increasing vascular stiffness and therefore the force exerted on the vessels (66). Gender also influences prevalence, with females at higher risk of onset than men (67, 68). Lifestyle factors, namely high BMI and being a smoker are both positively associated with hypertension (69, 70). Lastly, the offspring of hypertension positive parents are at increased risk of onset (19). Comparably, several studies have designed hypertension risk prediction models, associating the discussed variables with increased prevalence. Notably, Zhao et al. (71) found BMI, age, and family history as the primary risk factors, while Sun et al. (72) performed a comprehensive review, stating BMI, age, smoking status, blood pressure and parental history as established and traditional factors. Furthermore, Parikh et al. (73) computed an accurate risk prediction model concluding significance in all associated variables. The extensive research and similarities between literature indicate the health assessment is well-founded and viable.

##### 4.2.3.2 Mental stress

The mental stress insight reflects an individual's mental state and ability to deal with the stresses of life (74). Stress is a response to change, implemented with an overall goal to maintain stability and homeostasis. It manifests as a rise in the sympathetic nervous system activity, often causing physical, mental and behavioural disruption (73). Previous research suggests that this autonomic imbalance can be accurately computed by measuring HRV, the variance in rate between successive heartbeats (75–77). While SNS activity elevates HR, the reduction in PNS activity supresses beat-to-beat variability, suppressing HRV (75). Therefore, this relationship could indicate HR and HRV as ideal tools to accurately reflect stress levels (75, 78). Additionally, various studies have previously explored this relationship by evaluating rPPG as a method for stress detection, further emphasising the validity of rPPG-based stress detection (16, 48, 49). Notably, a study by Fontes et al. (16) successfully concluded high accuracy of rPPG for stress detection, with 95.83% of samples correctly classified into stress or non-stress categories relative to ground truth values derived from a PPG device. The paper also names HRV as an accurate indicator of stress levels, suggesting a prior basis for the applicability of rPPG in detecting stress.

Consequently, the insight can be computed by integrating rPPG measured HRV, HR and RR with the user inputs age, sex and stress levels. While HR and HRV have previously been validated as biomarkers for stress detection, the use of RR remains relatively underexplored. Nevertheless, foundational evidence suggests that its incorporation could further enhance detection. Nicolo et al. (79) supports this approach by addressing the strong association between RR and emotional stress, attributed to their partial regulation by the amygdala, a brain region involved with emotional processing. Considering the direct relationship between the biomarker and health derivation, this may be a valuable additional input for accuracy levels. Similarly, the model accounts for the influence of gender and age on stress levels and susceptibility. Studies show that women typically have higher vulnerability and prevalence to stress, suffering from higher rates of depression and anxiety. Symptoms are heightened and the exposure to chronic stressors is increased, all contributing to gender difference (80, 81). Age is also a critical factor, with stress susceptibility and perception tending to increase with age, resulting in higher levels among older adults (82). Considering their significant relevance to stress, their incorporation may further enhance the rPPG based detection.

##### 4.2.3.3 Mental health risk

The mental health risk is intended as an indicator to evaluate an individual's potential for developing a mental disorder over time. The review revealed several papers which highlighted the promise of rPPG in assessing mental health (50–54). Significantly, the biomarker HRV has been documented as useful for physicians to evaluate the level of depression a patient may be experiencing (50). Moshe et al. (52) explored the applicability of PPG signals for the detection of symptoms of anxiety and depression, achieving a high correlation between the technique and ECG for HRV assessment. The relationship between HRV and the disorders was also found to be significantly correlated (r = 0.98), demonstrating the method's applicability. Considering rPPG is the contactless version of PPG, the results can seemingly be translated between one another. Additionally, Dagdanpurev et al. (54) found finger-based PPG to be an accurate method of major depressive disorder, with a sensitivity of 83% and specificity of 93%. Facial video methods have also been found as reliable for assessing depression with a study by Unursaikhan et al. (51) demonstrating a sensitivity of 73% and specificity of 85%. Furthermore, a highly significant paper by Casado et al. (50) evaluated the usage of rPPG for assessing depression, and found it to be highly accurate with a mean average error of 7.44, and a root mean square error of 9.55. Evidently, prior research supports the adoption of rPPG in assessing mental health risk.

The integration of user reported data with stress based rPPG measurements may be beneficial for more robust health assessment. More specifically, mental stress history can be recorded over three weeks. Various studies have shown how stress is able to predict several types of mental illness, often acting as a risk factor for the development of depression associated disorder (74, 80). Montgomery et al. (83) also sets precedence for the three week period by identifying persisting, chronic stress as a major contributor to mental health disorders. Considering this, the input of stress history could be highly significant to enhance detection accuracy (83). Furthermore, as discussed HRV is one of the inputs for mental stress, directly impacting the mental health risk. Mulchay et al. (84) describes this relationship as highly impactful, with HRV serving as an indicator of health quality and disease. Mulchay details its dysregulation as directly correlated to intrusive thinking and mental health disorders, with values decreasing in the presence of related symptoms. The imbalance often indicates a disruption of inhibitory control within the brain, as the brain can no longer suppress mood related disturbances. Given the established association between a lower HRV, higher stress levels and various mental health disorders, this makes its computation from rPPG likely substantial.

Furthermore, the applicability of questions is highly relevant for assessing mental status, with questionnaires such as the GAD-7 (85) and PSS-10 (86) serving as the clinical standard. Prior research has demonstrated these questionnaires as highly relevant and sensitive for mental health detection (87–89). Notably, Löwe et al. (87) evaluated the validity of the GAD-7 in the general population, concluding it to be highly reliable. Questions should target the key hallmarks of mental disorder, with questions focusing on chronic stress, negative emotions, coping mechanisms and vulnerability (90).

##### 4.2.3.4 Energy balance

The aim of this insight is to assess the equilibrium of our autonomic nervous system (ANS). The review identified several studies evaluating the suitability of rPPG and PPG for measuring ANS balance and activity (24, 55–57). A conference paper by Constantino et al. (55) proposed a method to extract pulse rate variability, and therefore ANS status using rPPG. The paper reasons that the significant role of the ANS in affecting HR and HRV can be leveraged to reflect autonomic balance. A study by A van Es et al. (24) further supports the applicability of rPPG for ANS assessment, considering the ability of the technique to reliably extract pulse rate variability features in both the time and frequency domains. The applicability of PPG was further demonstrated through its identification of four ANS states: baseline, parasympathetic nervous system activation, peripheral SNS activation, cardiac SNS activation. Baseline served as a control state for the comparison with the activated ANS conditions. The method showed strong potential, reaching accuracies of 0.93, 0.98 and 0.84, respectively (56). Additionally, the detection of rPPG based HR and HRV has been widely explored, achieving significantly high accuracy levels (3, 11, 89). Cumulatively, these studies demonstrate a solid foundation and precedent for the relevance of rPPG in measuring HRV metrics, ANS function and energy balance.

Energy balance derivation may be based on the HRV metrics, Baevsky's stress index (BSI), the average interbeat interval (IBI) and the standard deviation of normal to normal (SDNN). The ANS is composed of both the parasympathetic nervous system and the SNS, with the former responsible for rest and digest functions, while the latter for flight or fight response. Prior research states that healthy individuals typically have balanced systems, with imbalances primarily due to disturbances, such as stressful events (91). These fluctuations can be measured and leveraged to assess energy levels. Kim et al. (78) found that higher SNS activity indicates psychological and physiological stress, an imbalance which affects HRV values. Similarly, Odinaev et al. (91) and (92) found BSI, IBI and SDNN to be reliable measures of SNS activity, and therefore of ANS imbalances. The BSI directly signifies SNS activity, for which higher values indicate higher stress levels. The IBI is inversely correlated with HR, as HR increases due to stress, the IBI decreases. This means that longer IBI is associated with parasympathetic activity, while shorter IBI reflects SNS activity. Similarly, SDNN values decrease as SNS activity increases, with lower values corresponding to a decrease in HRV in the presence of stress. The relationship between these markers and their significant relevance to ANS activity indicates a potential applicability of rPPG in energy balance computation.

##### 4.2.3.5 Sleep quality

This metric is a reflection on general sleep health, including sleep duration and sleep-wake patterns. It also assesses the quality of sleep experienced the previous night, providing insights into recovery and overall health. The correlation between rPPG measurements and sleep has previously been documented by Van Meulen et al. (58), who applied rPPG on sleeping patients in the dark to classify sleeping stages. The technique achieved high accuracy, with 81% of measurement assigned to the same 3-class sleep stages as polysomnography manual scoring. Kotzen et al. (59) confirms PPG's suitability in measuring sleep staging, with their model having achieved a Cohen's Kappa of 0.75, highlighting good agreement between PPG and standard methods. Additionally, HRV based PPG has been successfully applied as a method for fatigue detection, with 97.63% of predictions aligning with ground-truth observer rated labels (60). Although previous research has not directly applied rPPG for sleep quality assessment, these findings highlight its potential and relevance in sleep-based evaluations.

The combination of energy balance measurements, facial key-point detection and questions may provide a more comprehensive assessment of sleep quality. As discussed, the energy balance insight can be derived to provide a reflection on ANS regulation, which has a direct relationship with sleep quality. Research has shown that higher SNS activity is linked to poorer sleep conditions, including difficulty with falling asleep (93). Theoretically, this hyperactivation could be detected by the energy balance insight as an increase in BSI and a decrease in HRV, the SDNN and the IBI. Facial key-points in the form of drooping/hanging eyelids, paler skin, redder eyes, more puffy/swollen eyes, dark circles under the eyes, more wrinkles/fine lines and more drooping corners of the mouth are also considered to determine a more comprehensive measure (60). Previous studies have highlighted the efficiency of facial landmarks for sleep and fatigue detection, documenting high accuracies of 97.63% (60) and 98.6% (94). Notably, Yu et al. (60) combines facial features and PPG signals to build a robust model. In addition to the rPPG measurements, the incorporation of self-reported data, could further enhance assessment. Past literature has documented the efficiency of questionnaires, more specifically the Pittsburgh sleep quality index (95). Additionally, sleep latency and disturbances, two key components in sleep quality (96) should be considered.

## 5 Limitations

As with any research, several limitations were encountered, including constraints in the literature review methodology. Firstly, the review was limited to English-written. Although not considerably, this decreased the research paper sample size, a limitation which could have been overcome with the use of translation services. Concerning the search strategy, the choice of limited terms combined with a highly restrictive screening process may have omitted relevant, foundational studies. The review limited search terms to “rPPG”, “remote photoplethysmography”, “PPG”, “iPPG” and “Non contact PPG” whereas important research may be published under alternative terms. This likely limited the output of studies in the review. The decrease in sample size could have limited significance, but also may have challenged the perception that no prior work has comprehensively assessed the broad potential of rPPG. Additionally, this may have contributed to a selection bias of overly positive findings, only representing the advantages of rPPG. Bias may have also occurred on the basis of only favoring the selection of “newer” papers. The motivation by IntelliProve may be subject to a conflict of interest, given the author's relation to the company, however all efforts have been made to remain critical and objective. Lastly, the combination of rPPG technology with user inputs, including questionnaires and the assessment of micro-expression to derive health outcomes introduces enhanced input, which may present rPPG in an overly favorable light.

Furthermore, rPPG faces several drawbacks which limit the technology's application and accuracy. Primarily, the technology is sensitive to motion artefacts and lighting, requiring individuals to remain static and in stable lighting during assessment. Variability can affect signal accuracy, leading to measurement errors (30). Skin tone and tissue properties, namely darker complexions and variability in skin reflectance further reduce the technology's ability to acquire a reliable signal (31). Additionally, current applications of rPPG focus on assessing general well-being, rather than detecting specific pathologies. This is especially relevant in the mental health field, where the complexity of the SNS may expose the assessment to significant bias. For the purpose of this review, the abovementioned limitations must be addressed and adequately controlled for. This is particularly crucial for the nature of this paper, which advocates the usage of rPPG for health assessment. This review has attempted to mitigate these limitations by proposing a combined system, integrating rPPG with other reliable methods such as facial landmark detection and questionnaires. Despite this, further improvements could be made by advancing signal processing algorithms, and by integrating additional modes into the system. Further research into combined systems is necessary.

## 6 Conclusion

This review assesses the potential of rPPG in deriving health insights. Despite rPPG being reported in literature as a revolutionary tool to measure physiological markers, there is a gap of knowledge in regard to its synthesised applicability. Heart rate, respiration rate and heart rate variability are widely accepted and validated measures performed by rPPG, whereas literature on further health insights is emerging. Stress detection levels and hypertension prevalence have been briefly explored, but more research is warranted on the use of the technology. Using a combination of optimised rPPG software and user inputs, the review was able to identify several promising health insights, namely mental health risk, energy balance, sleep quality and the resonant breathing score. This review hopes to set the groundwork for future research in the field, by exploring further applications of rPPG. Evidently, the technology shows considerable potential in the digital health field, warranting the need for continued research on rPPG and its full potential.
