# Supplementary material for: Remote photoplethysmography for health assessment: a review informed by IntelliProve technology
Source: Front Digit Health. 2026 Jan 5;7:1667423. doi: 10.3389/fdgth.2025.1667423 (PMC12812591; doi:10.3389/fdgth.2025.1667423)
Supplement: Supplementary file 1 [file Table1.pdf]

Supplementary Material

1 Supplementary Table

Table 1. Overview of the included studies, showing their individual characteristics.

**Author (Year)**	**Type of Paper**	**Technology Type**	**Usage Focus**
Pirzada et al. (2024)	Review	rPPG	Overview of rPPG techniques for heart rate and blood oxygenation measurement
Di Lernia et al. (2024)	Research paper	rPPG	Remote heart rate imaging with webcams; data for real-world rPPG application
Gudi et al. (2020)	Research paper	rPPG	Real-time heart rate and HRV estimation using webcam data
Lee et al. (2023)	Review	rPPG	Review of remote heart rate measurement using PPG
Salim & Khidhir (2024)	Review	rPPG	Comprehensive review of rPPG methods for heart rate estimation
Allado et al. (2022)	Research paper	rPPG	Validation of respiratory rate measurement using remote PPG in clinical setting
Qiao et al. (2022)	Research paper	rPPG	Smartphone camera-based remote vital signs measurement
Tiwari et al. (2019)	Conference paper	NA	Breathing rate complexity features for stress measurement
McDuff et al. (2014)	Research paper	rPPG	Remote detection of systolic and diastolic peaks via digital camera

Umair et al. (2021)	Research paper	PPG	Comparison of wearable heart rate sensors for HRV biofeedback
Shaffer & Ginsberg (2017)	Review	NA	Overview of HRV metrics and normative data
Lee et al. (2024)	Research paper	NA	Respiratory rate monitoring assessment in emergency department
Drummond et al. (2020)	Research paper	NA	Clinical methods assessment of respiratory rate measurement precision
Kim (2023)	Review	NA	Arterial stiffness and hypertension overview
Rauniyar et al. (2020)	Research paper	PPG	Oxygen saturation study in ICU patients
Fontes et al. (2024)	Research paper	rPPG	Stress detection using rPPG analysis and deep learning
Curran et al. (2023)	Review	rPPG	Camera-based blood pressure measurement overview
Fukunishi et al. (2018)	Conference paper	rPPG	Video-based HR and HRV spectrogram measurement
Stamler et al. (1979)	Research paper	NA	Study on family history and hypertension prevalence
Elgendi et al. (2024)	Research paper	rPPG	Signal quality index optimization for remote PPG sensing
Haugg et al. (2022)	Research paper	rPPG	Analysis of remote PPG construction methods
Boccignone et al. (2020)	Research paper	rPPG	Framework for rPPG methods and assessment

Wang et al. (2017)	Research paper	rPPG	Algorithmic principles for remote PPG
Es et al. (2023)	Research paper	rPPG	Contactless cardiovascular assessment comparison
Momeni et al. (2024)	Conference paper	rPPG	Facial rPPG during cold liquid bolus administration
Rao et al. (2025)	Research paper	rPPG	Long-distance heart rate measurement via POS method
Kwon et al. (2015)	Conference paper	rPPG	ROI analysis for facial video rPPG
Kim et al. (2021)	Research paper	rPPG	ROI selection assessment for facial rPPG
Bondarenko et al. (2025)	Research paper	rPPG	Role of face regions in contactless heart rate monitoring
Debnath & Kim (2025)	Review	rPPG	Review of heart rate measurement using rPPG and deep learning
Dasari et al. (2021)	Research paper	rPPG	Biases evaluation in rPPG methods
Zuccotti et al. (2025)	Research paper	rPPG	Accuracy evaluation of heart rate, oxygen saturation and blood pressure via non-contact PPG
Shoushan et al. (2021)	Research paper	rPPG	Non-contact heart rate monitoring during respiratory maneuvers and body movements
la Cruz et al. (2021)	Research paper	rPPG	Simultaneous instantaneous heart and respiratory rate estimation
Farahani et al. (2024)	Research paper	rPPG	Non-contact imaging of the neck for cardiopulmonary monitoring

Rodriguez Berrío & Ramos-Castro (2018)	Research paper	rPPG	Video pulse rate variability analysis under stationary and motion conditions
Wei et al. (2017)	Research paper	rPPG	Non-contact synchronous dynamic measurement of respiratory and heart rate
Romero et al. (2024)	Conference paper	PPG	Earable-based PPG system for continuous respiratory monitoring
Jan et al. (2019)	Research paper	PPG	Coherence evaluation between ECG and PPG for HRV and respiration
Cheng et al. (2025)	Conference paper	rPPG	Remote blood pressure estimation using facial videos
Schrumpf et al. (2021)	Research paper	PPG/rPPG	Non-invasive blood pressure prediction from PPG and rPPG
Putten & Bamford (2023)	Conference paper	rPPG	Systolic blood pressure prediction from remote PPG
Trirongjitmoah et al. (2024)	Research paper	rPPG	Heart rate and blood pressure estimation from image photoplethysmography
Fang et al. (2024)	Research paper	rPPG	Fair non-contact blood pressure estimation
Park & Hong (2024)	Research paper	rPPG	Robust blood pressure measurement from facial videos
Liang et al. (2018)	Research paper	PPG	Hypertension risk stratification using photoplethysmography
Liang et al. (2018)	Research paper	PPG	Hypertension assessment via PPG
Morales-Fajardo et al. (2022)	Research paper	rPPG	Stress identification using rPPG in academic environments

Lee et al. (2024)	Research paper	rPPG	Ultra-short-term stress measurement using camera-based rPPG
Álvarez Casado et al. (2023)	Research paper	rPPG	Depression recognition from facial video rPPG
Unursaikhan et al. (2021)	Research paper	PPG	Webcam-based major depressive disorder screening system
Moshe et al. (2021)	Research paper	Wearables	Predicting depression and anxiety symptoms using smartphone and wearable data
Lyzwinski et al. (2023)	Review	PPG	Use of PPG in mental health assessment
Dagdanpurev et al. (2018)	Research paper	PPG	Autonomic response-based MDD screening system using fingertip PPG
Constantino et al. (2021)	Conference paper	rPPG	Real-time pulse rate variability for remote autonomic assessment
Liu et al. (2021)	Research paper	PPG	Autonomic nervous system assessment based on PPG
Kim et al. (2022)	Research paper	PPG	Real-time cardiovascular and autonomic nervous system analysis using PPG signals
van Meulen et al. (2023)	Research paper	Camera PPG	Contactless camera-based sleep staging
Kotzen et al. (2023)	Research paper	PPG	Deep learning for sleep staging from continuous PPG fibers
Yu et al. (2024)	Research paper	PPG	Driver fatigue detection using PPG and facial features
Avram et al. (2019)	Research paper	NA	Real-world heart rate norms in large cohort

Olshansky et al. (2022)	Review	NA	Importance of resting heart rate
Tanzmeister et al. (2022)	Research paper	NA	Resonance frequency breathing to reduce cardiovascular stress
Chaitanya et al. (2022)	Research paper	NA	Effects of resonance breathing on HRV and cognitive functions
Singh et al. (2019)	Research paper	NA	Prevalence and risk factors for hypertension
Vasan et al. (2001)	Research paper	NA	Frequency of progression to hypertension
Bakx et al. (1999)	Research paper	NA	Blood pressure development and hypertension incidence
Dyer et al. (1999)	Research paper	NA	Incidence and predictors of elevated blood pressure
Wilsgaard et al. (2000)	Research paper	NA	Impact of body weight on blood pressure
Hubert et al. (1987)	Research paper	NA	Lifestyle correlates of coronary heart disease risk factors
Zhao et al. (2021)	Research paper	NA	Hypertension risk prediction using machine learning
Sun et al. (2017)	Systematic review	NA	Risk prediction models for incident hypertension
Parikh et al. (2008)	Research paper	NA	Risk score for predicting hypertension incidence
WHO (2022)	Fact sheet	NA	Mental health data and response

Peabody et al. (2023)	Systematic review	NA	HRV as a stress measure in medical professionals
Grässler et al. (2021)	Research paper	NA	Resting HRV and sleep quality in mild cognitive impairment
Attar et al. (2021)	Research paper	NA	Stress analysis using HRV and EEG
Kim et al. (2018)	Meta-analysis	NA	Stress and HRV literature review
Nicolò et al. (2020)	Review	NA	Importance of respiratory rate monitoring
Mayor (2015)	Review	NA	Gender roles in stress and health
Sandanger et al. (2004)	Research paper	NA	Women's mental health susceptibility to stress
Osmanovic-Thunström et al. (2015)	Research paper	NA	Perceived stress levels and age
Montgomery et al. (2024)	Research paper	NA	Impact of chronic stress on physical and mental health
Mulcahy et al. (2019)	Review	NA	HRV as biomarker in health and affective disorders
Spitzer et al. (2006)	Research paper	NA	GAD-7 anxiety assessment tool
Kroenke et al. (2001)	Research paper	NA	PHQ-9 depression severity measure
Löwe et al. (2008)	Research paper	NA	GAD-7 validation in general population
Lee (2013)	Erratum	NA	Correction to Perceived Stress Scale review

Baik et al. (2019)	Research paper	NA	Perceived Stress Scale-10 reliability in Hispanic Americans
Mohr et al. (2014)	Research paper	NA	Coping behavior under chronic stress and psychiatric disorder vulnerability
Odinaev et al. (2023)	Research paper	rPPG	Camera-based HRV and stress measurement from facial videos
Odinaev et al. (2023)	Research paper	rPPG	Robust HRV measurement from facial videos
Oliver et al. (2020)	Research paper	NA	Relationship between sleep and autonomic health
Jayakumari et al. (2024)	Research paper	NA	Drowsiness and sleep detection using facial landmark algorithm
Buysse et al. (1989)	Research paper	NA	Pittsburgh Sleep Quality Index (PSQI) for psychiatric research
Ohayon et al. (2017)	Research paper	NA	National Sleep Foundation sleep quality recommendations
IntelliProve (2024)	Validation report	rPPG	Device validation report for IntelliProve software medical device
